# A Systematic Review on the Prevalence of Tick‐Borne Encephalitis Virus in Milk and Milk Products in Europe

**DOI:** 10.1111/zph.13216

**Published:** 2025-02-23

**Authors:** Laura Tomassone, Elisa Martello, Alessandro Mannelli, Aurora Vicentini, Céline M. Gossner, Jo Leonardi‐Bee

**Affiliations:** ^1^ Department of Veterinary Sciences University of Turin Grugliasco Italy; ^2^ Centre for Evidence Based Healthcare, School of Medicine University of Nottingham Nottingham UK; ^3^ European Centre for Disease Prevention and Control (ECDC) Solna Sweden

**Keywords:** alimentary, dairy, ruminants, TBEV, transmission, unpasteurized

## Abstract

**Background:**

Tick‐borne encephalitis virus (TBEV) is one of the most significant zoonotic diseases in Europe. It primarily spreads through the bites of infected ticks and, less frequently, through consumption of raw milk and dairy products from viremic domestic ruminants.

**Aims:**

Assess the prevalence of TBEV or anti‐TBEV antibodies in milk and milk products from domestic ruminants in Europe.

**Materials and Methods:**

Systematic literature review adhering to the JBI methodology, and reported following the PRISMA framework.

**Results:**

From the 16 included scientific articles, we extracted 35 data collections (31 on raw milk and 4 on raw milk cheese); studies focused on cow (*n* = 15), goat (*n* = 11) and sheep milk (*n* = 5), goat (*n* = 3) and cow/goat cheese (*n* = 1). Fifteen data collections involved individual milk and 16 bulk milk samples. The estimated prevalence of TBEV in individual raw milk and cheese was 6% and 3%, respectively. TBEV prevalence in bulk milk was very heterogeneous, with most values either 0% or 100%.

**Discussion:**

Although published research on TBEV transmission to humans through milk and dairy products in the EU countries is limited, our results highlight the potential infection risk for consumers. The variable prevalence reported in the studies may reflect the focal nature of TBEV.

**Conclusion:**

Studies on unpasteurised dairy products from domestic ruminants can be valuable for the detection of TBEV presence in a geographic area, even when human cases are not reported. Thanks to the ease of sample collection, their testing could be adopted in monitoring plans on TBEV.

**Summary**

- Alimentary outbreaks of TBEV occur in Europe, but published information on the prevalence of TBE virus/antibodies in dairy products is limited.
- TBEV prevalence reported in milk and products is variable, reflecting the focal nature of TBEV.
- The ease of obtaining dairy products and conducting tests on them can aid in risk assessment and contribute to TBE epidemiological surveillance.

## 1 Introduction

Tick‐borne encephalitis virus (TBEV) is classified within the Flavivirus genus and is responsible for one of the most significant zoonotic diseases in Europe, with its incidence and geographic range on the rise in recent years (ECDC 2022). TBEV primarily disseminates through the bites of infected ticks, with *Ixodes ricinus* and *Ixodes persulcatus* being the primary vectors (Estrada‐Peña and de la Fuente 2014). However, an intriguing aspect of TBEV transmission is its potential to be contracted through the consumption of raw milk and dairy products derived from domestic ruminants that are viremic (Ličková et al. 2022; Martello et al. 2022). Numerous studies have extensively explored TBEV seroprevalence in domestic ruminants, particularly goats and sheep, while investigations involving cows are comparatively less frequent (Springer et al. 2020). Upon TBEV infection, domestic ruminants typically develop asymptomatic viremia, and it is during this viremic phase that animals excrete the virus in their milk, thereby posing a potential risk for human infection through the consumption of raw dairy products. Among domestic animals, goats are most commonly infected with TBEV. This might be due to their way of grazing, browsing preferentially brushes and wood edges, which are very suitable for questing ticks (Ličková et al. 2022). In addition, goats can be infected multiple times throughout their lifespan, excreting the virus in their milk for an extended period, detectable between 3 and 25 days post‐infection, as reported in experimental studies (reviewed by Ličková et al. 2022; Balogh et al. 2012). Studies on infected sheep have shown that TBEV can be detected in milk for a period ranging from 2 to 7 days, with a peak viral load occurring on the 5th day (reviewed by Ličková et al. 2022). There is limited information available regarding TBEV infection in cows. Studies have indicated that TBEV can be found in the milk of infected cows from day 2 to day 8 after infection (Salat and Ruzek 2020). Nevertheless, laboratory investigations have revealed that the viral loads in cows do not reach the levels observed in sheep and goats.

The dual transmission route (through vectors or via alimentary) highlights the complexity of TBE epidemiology and underscores the importance of a multifaceted approach to its surveillance and control. While ticks remain the primary vector, the role of viremic domestic ruminants in the transmission cycle cannot be underestimated (Martello et al. 2022; Springer et al. 2020), making it essential to investigate this lesser‐known route of infection. Thus, the detection of TBEV in dairy products could be an additional indicator in TBE surveillance plans, combined with data collection from other sources (people, questing vectors and animal hosts).

We conducted a systematic review to assess the prevalence of TBEV in milk and milk products from domestic ruminants in Europe, and to evaluate the usefulness of monitoring TBEV infection in dairy products for the early identification of viral circulation in a geographic area.

## 2 Material and Methods

This systematic review was conducted and reported adhering to the JBI methodology for systematic reviews (Aromataris and Munn 2020) and the PRISMA (Preferred Reporting Items for Systematic reviews and Meta‐Analyses) framework (Moher et al. 2009), respectively. The protocol for this review entitled ‘Tick‐borne encephalitis infection in milk and dairy products from domestic ruminants in Europe: a systematic literature review’ was published in PROSPERO (CRD42021279317) (Data S1). Available from: https://www.crd.york.ac.uk/prospero/display_record.php?ID=CRD42021279317.

### 2.1 Inclusion Criteria

We included studies which performed any laboratory test on milk or cheese or other dairy products (yogurt, butter, cream, ice‐cream) to identify TBEV or anti‐TBEV antibodies and which provided numerical data. Reviews, letters, congress abstracts, and opinion pieces were excluded. We narrowed the search to the 27 member countries of the European Union (EU), Iceland, Norway, Switzerland, and the UK (Data S2).

### 2.2 Search Strategy and Study Selection

We conducted thorough electronic searches of Medline, EMBASE, and CAB Abstracts (from the beginning until 01/11/2022) (Data S3). Additionally, we reviewed the reference lists of included papers and previous reviews to find additional relevant studies. We screened and included studies irrespective of language. Two reviewers independently screened the titles and abstracts, and in case of any discrepancies, a third reviewer was involved in the decision‐making process. Full texts of potentially eligible studies and studies without available abstracts were obtained and independently screened by two reviewers, with any disagreements resolved by a third reviewer.

### 2.3 Data Extraction and Critical Appraisal

Two reviewers independently conducted data extraction and critical appraisal of the methodological quality for all the included studies. Standardised data extraction and JBI critical appraisal (available at https://jbi.global/critical‐appraisal‐tools) were utilised in this process to assess the trustworthiness, relevance and results of the included studies.

In situations where the samples were tested during a human TBEV outbreak investigation, the case series JBI tool was employed, but questions 4, 7 and 8 were not applicable. Conversely, when the paper described the prevalence of TBEV in milk or milk products, the JBI prevalence tool was utilised, but question 9 was not applicable. Multiple data collections could be present in the same study, for example, data on different animal species (goat, sheep, cow), type of sample (individual or bulk milk, cheese batch), sampling period (year, month). In this case, we used only one data collection type as the basis for the critical appraisal assessment. Each question was assigned either ‘yes’ (1 mark), ‘no’ (0 marks), ‘unclear’ (0.5 marks) or ‘not applicable (NA)’. A total score was also calculated. Answers ‘NA’ were not included for assigning the score. High quality was given where all questions were answered as ‘yes’; moderate was given where the answers were either ‘yes’ or ‘unclear’; low was given where at least one answer was ‘no’.

### 2.4 Data Synthesis

The characteristics of the included studies are presented in a tabular format, providing information on the study design, geographical location, type of sample, sample size, animal species, setting, diagnostic test and main findings of each study. Initially, a narrative synthesis approach was employed, utilising content analysis to systematically describe and summarise the included studies. The studies were categorised based on geographical location, year of data collection, animal species and the samples tested.

We calculated raw proportions using the number of events divided by the total number of samples tested. Variances of the raw proportions were stabilised using the Freeman‐Tukey variant of the arcsine square root transformation to allow the inclusion of the whole range of proportions (0%–100%) (Stuart and Ord 1994). To estimate the prevalence of TBEV (evaluated by RT‐PCR), a meta‐analysis on the transformed quantity was conducted using a random effects model using the ‘metprop’ command in STATA (version 18) (StataCorp. 2023). Separate meta‐analyses were carried out based on the type of sample collected, which included individual milk and cheese batches. Whenever feasible, subgroup analyses were performed to examine variations in prevalence among products from different animal species.

Heterogeneity was quantified using *I* ^2^ statistics (Higgins et al. 2003). Due to a small number of studies, we were unable to conduct a planned subgroup analyses or publication bias assessment.

## 3 Results

### 3.1 Study Selection

**FIGURE 1:**
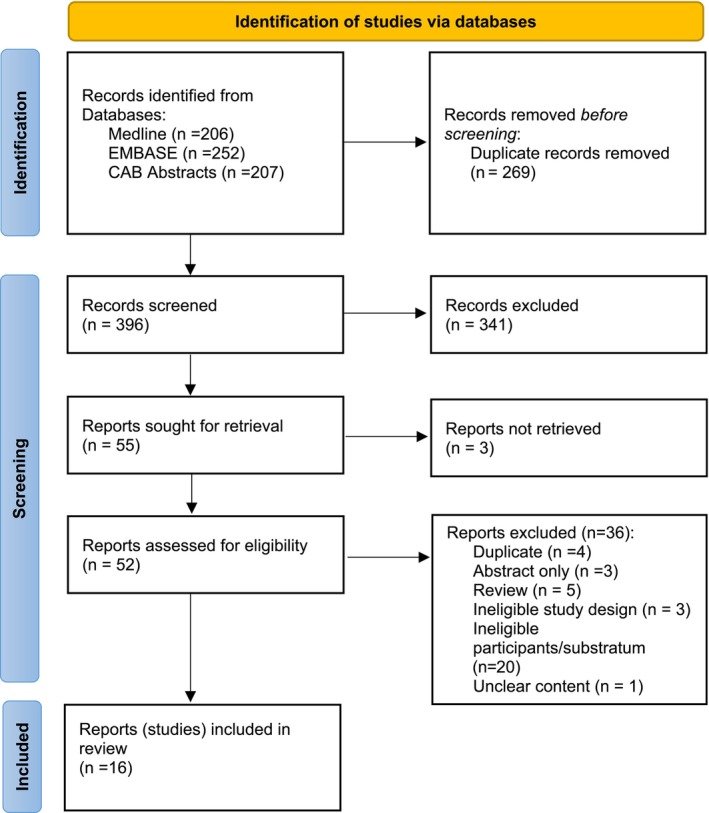
PRISMA Flow Chart of included studies.

The searches identified 665 results, of which 269 were duplicates. A total of 341 were excluded following title and abstract screening, and 55 were eligible for full text screening. Of these, we did not retrieve three full texts. We included 16 papers and excluded 36 (PRISMA flow chart, Figure 1, and Data S4). A total of four papers included in the review required translation into English from Hungarian (*n* = 1), German (*n* = 1) and Czech (*n* = 2).

The majority of data collections investigated TBEV infection in goat samples (*n* = 11), then cow (*n* = 6) and sheep (*n* = 5). Only one study tested cheese made from a mixture of goat and cow milk. Ten data collections investigated TBEV in bulk milk, nine in individual milk, two in both bulk and individual milk, and four in batch cheese (one of those tested also bulk milk).

Most of the studies were conducted in Austria (2), Croatia (2), Slovakia (2), Sweden (2), then Czechia (1), France (1), Germany (1), Hungary (1), Lithuania (1), Norway (1), Poland (1), and Slovenia (1) (Table 1).

**TABLE 1:** Characteristics of included studies (grouped by animal species).

Publication ID	Country	Year of data collection	Months of data collection	Sample tested	Type of study
Cow					
Malena et al. 2014	Czechia	2014	July–September	Bulk milk	Prevalence
Gonzalez et al. 2022	France	2020	June	Bulk milk	Outbreak investigation
Caini et al. 2012	Hungary	2011	November	Individual milk	Outbreak investigation
Paulsen et al. 2019	Norway	2014–2017	June‐ Oct	Bulk and individual milk	Prevalence
Cisak et al. 2010	Poland	NA	NA	Individual milk	Outbreak investigation
Blomqvist et al. 2021	Sweden	2013	May, November	Bulk milk	Prevalence
Goat					
Holzmann et al. 2009	Austria	2008	July	Individual milk	Outbreak investigation
Mylonaki et al. 2022	Austria	NA	Summer	Bulk milk	Outbreak investigation
Markovinović et al. 2016	Croatia	2015	Spring–Summer	Batch cheese	Outbreak investigation
Ilic et al. 2020	Croatia	2019	July	Individual milk	Outbreak investigation
Malena et al. 2014	Czechia	2014	July–September	Bulk milk	Prevalence
Gonzalez et al. 2022	France	2020	June–July	Batch cheese	Outbreak investigation
Gonzalez et al. 2022	France	2020	June	Bulk and individual milk	Outbreak investigation
Pautienius et al. 2021	Lithuania	2018–2019	April, November	Bulk milk	Prevalence
Cisak et al. 2010	Poland	NA	NA	Individual milk	Outbreak investigation
Kohl et al. 1996	Slovakia	1993	NA	Individual milk	Outbreak investigation
Hudopisk et al. 2013	Slovenia	2012	Spring–Summer	Individual milk	Outbreak investigation
Brockmann et al. 2018	Germany	2016	June	Batch cheese, bulk milk	Outbreak investigation
Mixed Goat and Cow					
Holzmann et al. 2009	Austria	2008	July	Batch cheese	Outbreak investigation
Sheep					
Malena et al. 2014	Czechia	2014	July–September	Bulk milk	Prevalence
Pautienius et al. 2021	Lithuania	2018–2019	April, November	Bulk milk	Prevalence
Cisak et al. 2010	Poland	NA	NA	Individual milk	Outbreak investigation
Paraličová et al. 2022	Slovakia	2016	Spring–Summer	Bulk milk	Outbreak investigation
Wallenhammar et al. 2020	Sweden	2017	July	Individual milk	Prevalence

*Note*: Multiple data collections are also included when reported in the same manuscript.

The characteristics of included studies highlighting the animal species under investigation, the country where the study was conducted, the year of data collection, the months of data collection (when available), the diagnostic test employed, and the type of sample tested for each study are reported in Table 1.

A total of 10 studies described TBE outbreak investigations where samples from animals suspected to be the source of human infection were tested. In six studies, authors estimated the prevalence of TBEV in milk and/or milk products (Table 1).

In the included studies, the infection of TBEV was based on either the detection of antibodies in the milk or milk products through ELISA (enzyme‐linked immunosorbent assay; *n* = 3 studies), VNT (virus neutralisation test; *n* = 1 study), IFA (indirect fluorescent antibody test; *n* = 1 study), or the direct identification of the virus through RT‐PCR (reverse transcription polymerase chain reaction; *n* = 13 studies). Details of the diagnostic tests and the results are reported in Data S5.

### 3.2 Main Findings: Individual and Bulk Milk, Batch Cheese

We included studies carried out from 1993 to 2022, describing data collections performed from April to November, with most studies conducted in spring or summer. Testing of animal products followed human cases in outbreak investigations; in one case, a longitudinal study followed the outbreak investigation to monitor the animal serological status and virus excretion in milk after the establishment of control measures (Gonzalez et al. 2022). In prevalence studies (*n* = 6), sample collection and testing were performed at a single point in time in most cases; two articles reported repeated samplings at the same locations across the season to compare the antibody titres (Blomqvist et al. 2021; Pautienius et al. 2021).

Most studies focused on goat products (*n* = 11), followed by cow (*n* = 7) and sheep (*n* = 5) products (Table 1). Three types of samples were analysed: individual milk, bulk milk, and cheese. Cheese was analysed following human TBE outbreaks (*n* = 4 studies; Table 2), while milk was the object of both outbreak investigations and prevalence studies. Bulk milk was analysed in eight studies, including 16 data collections (studies on samples from different animal species, study areas or collection dates), while seven studies focused on individual milk samples, including 13 data collections (Table 1). As regards the employed diagnostic tests, all outbreak investigations adopted RT‐PCR as the test, except for the oldest study (Kohl et al. 1996), in which VNT was employed; in one outbreak investigation, both PCR and IFA were performed on the same samples, giving the same infection prevalence (Hudopisk et al. 2013). In prevalence studies, RT‐PCR was used as a single test in three studies, ELISA as a single test in two studies, and in one study, samples were tested with both PCR and ELISA (Data S5). In this last study (Cisak et al. 2010), the number of positive samples detected by PCR was higher compared to ELISA.

**TABLE 2:** Details of the cheese tested in batch for the direct detection of TBEV.

Publication	Country	Animal species	PCR type	Number tested	Number positives	Type of cheese	Details on fresh/ripened cheese as reported in the manuscript
Holzmann et al. 2009	Austria	Goat and Cow	RT‐PCR	3	0	Self‐made cheese prepared from a mixture of non‐pasteurised goat milk and cow milk	Fresh (‘The cheese was prepared from a mixture of fresh milk from 1 goat and 3 cows and was eaten shortly after production’)
Markovinović et al. 2016	Croatia	Goat	PCR	1	0	Raw goat cheese	Not specified
Brockmann et al. 2018	Germany	Goat	RT‐qPCR	22	5	Goat soft cheese (1/12); Goat cheese, ripened (1/2); Goat cream cheese (3/7); Goat cream cheese with curry (0/1) (number positives/number tested)	Fresh apart from 2 ‘ripened cheese’. PCR on unpasteurised goat cheese from the manufacturing and storage facilities of the dairy farm (16 June), and consumers provided available residual samples of cheese produced between 5 May to 15 June. Five samples from five different batches of cheese (cream cheese, soft cheese and ripened cheese) produced between 8 and 12 June tested positive by repeated RT‐qPCR. Two of the five samples could be confirmed by cell culture (goat cream cheese), indicating the viability of TBEV in the cheese
Gonzalez et al. 2022	France	Goat	RT‐PCR	91	7	Fresh goat cheese made of raw milk named ‘faisselle’	Fresh

**FIGURE 2:**
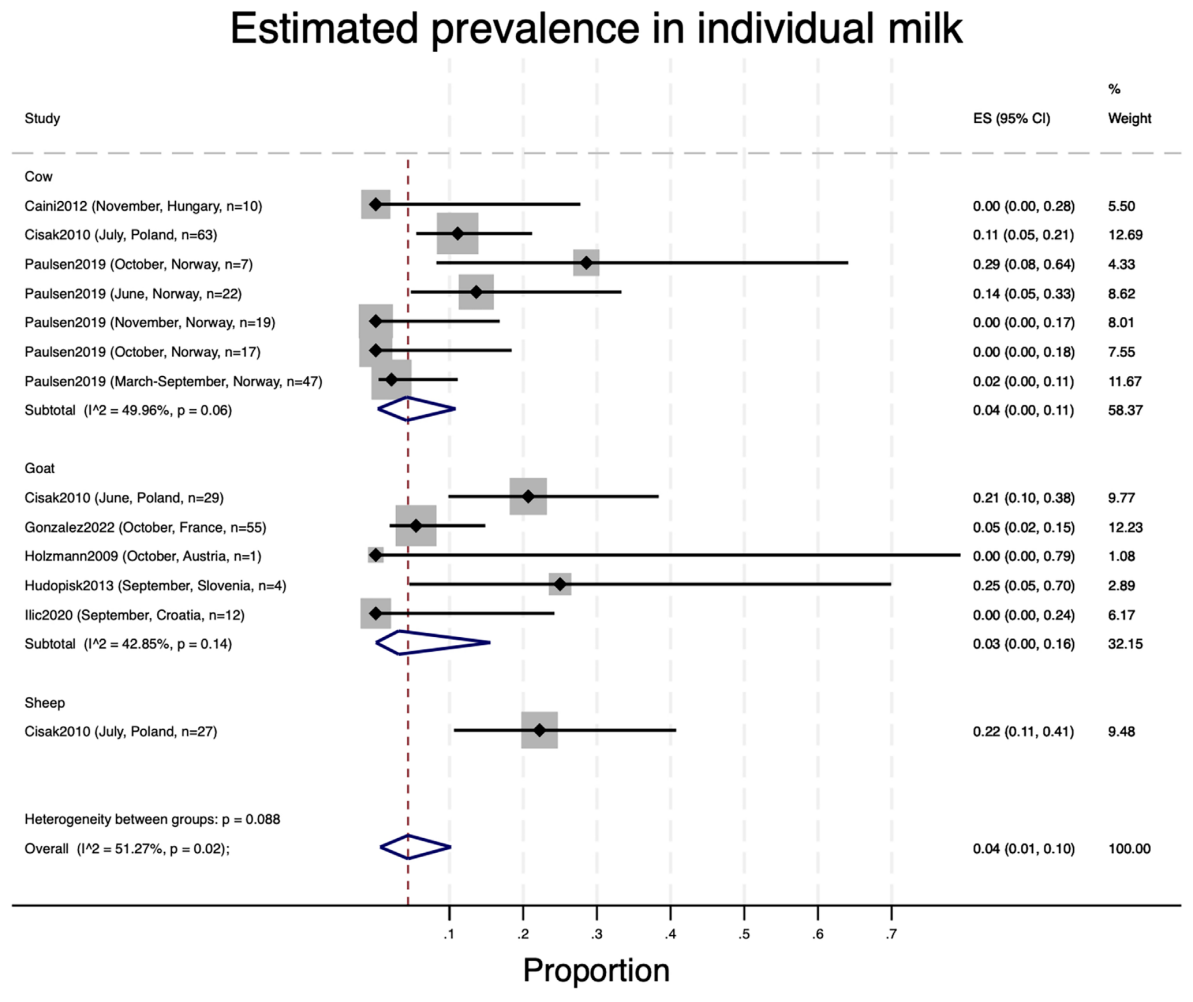
Pooled prevalence of TBEV in individual samples of raw milk. The red dotted line corresponds to the overall pooled estimate of prevalence.

The pooled prevalence of TBEV in individual raw milk samples was 6% (95% CI 1%–12%; Figure 2), with similar findings for cow milk (4%, 95% CI 0%–11%) and goat milk (3%, 95% CI 0%–13%); however, the prevalence was 28% (95% CI 15%–43%) in the one study in sheep milk. RT‐PCR was used for diagnosis in all studies except for two (Kohl et al. 1996; Wallenhammar et al. 2020). In the first, an outbreak investigation (Kohl et al. 1996), the individual milk samples from three goats owned by the patients tested negative by VNT. In the second, a prevalence study (Wallenhammar et al. 2020), only the ELISA test (confirmed by WB) was used to test individual sheep samples, which showed a 40% antibody prevalence (95% CI: 16%–68%).

**FIGURE 3:**
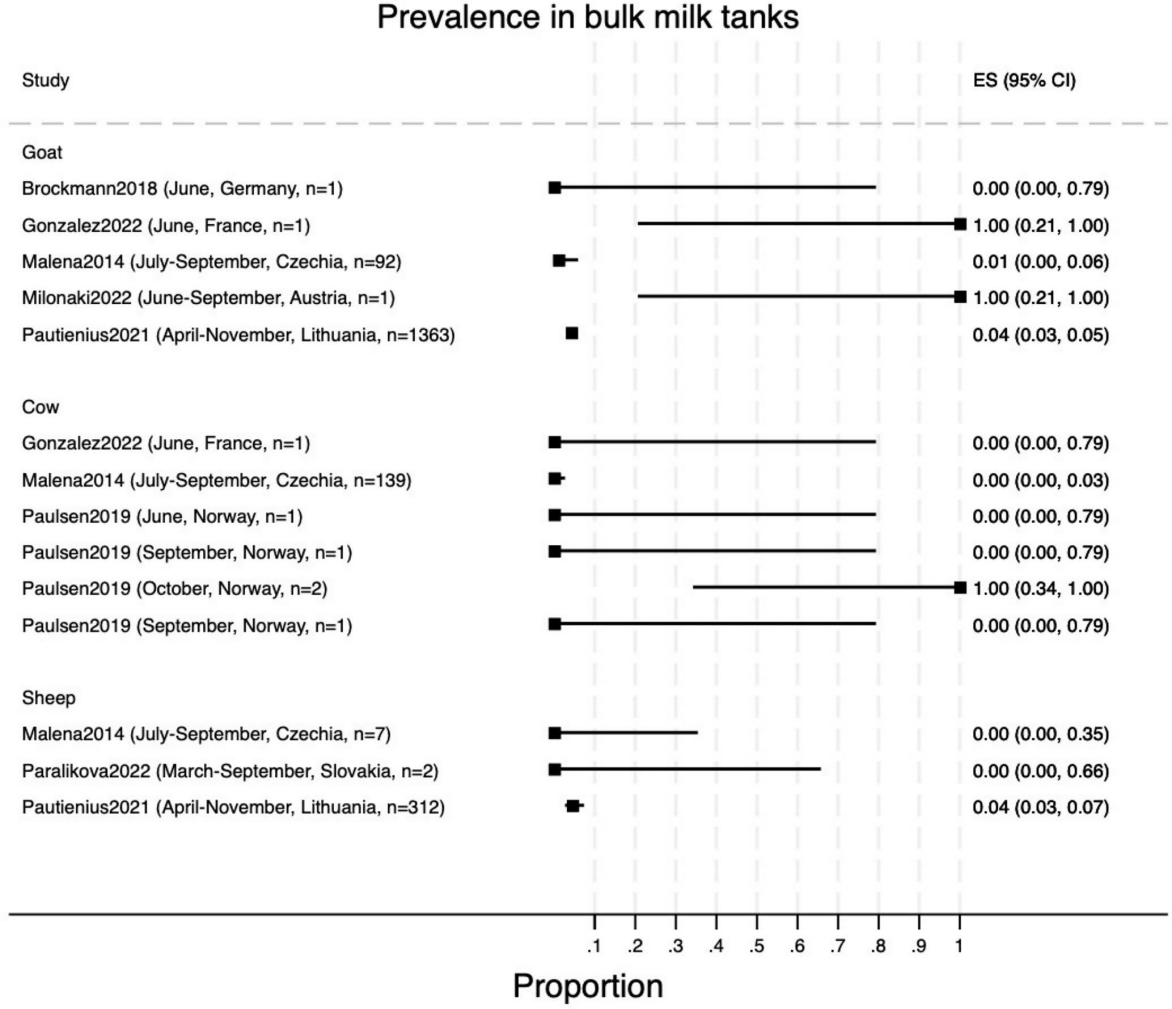
Prevalence of TBEV in bulk milk tank samples of raw milk.

The prevalence estimates for TBEV in bulk milk were very heterogeneous, with most values either 0% or 100% in studies testing only one or two samples; therefore, meta‐analysis was not conducted (Figure 3). RT‐PCR was used for diagnosis in all prevalence studies except one (Blomqvist et al. 2021), in which an ELISA test was applied on bulk tank milk collected from dairy herds before and after the vector season and reported a prevalence of 2.9% in May (95% CI: 1.7%–4.6%) and 4.8% in November (95% CI: 3.2%–6.9%).

**FIGURE 4:**
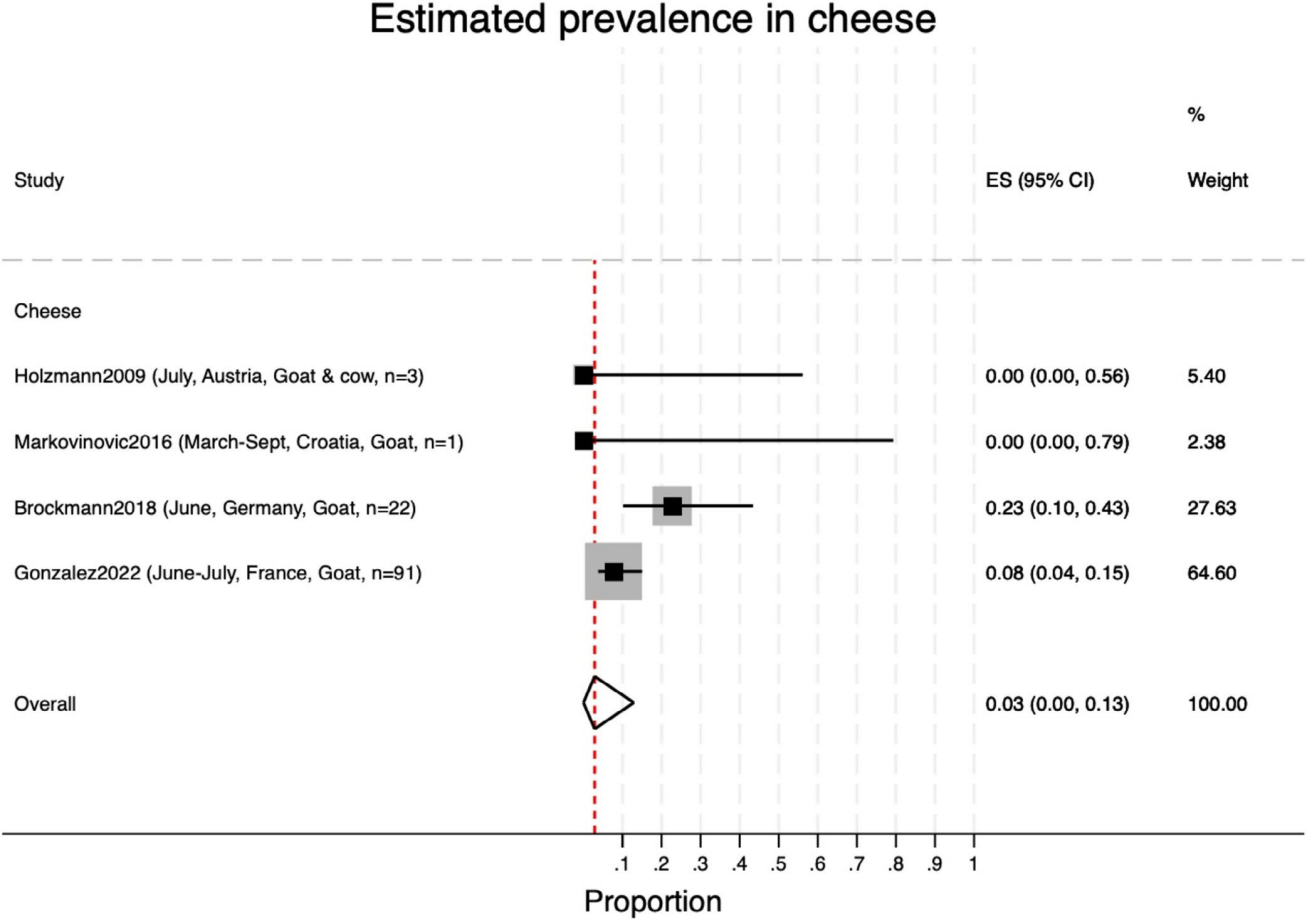
Pooled prevalence of TBEV in cheese made from raw milk. The red dotted line corresponds to the overall pooled estimate of prevalence.

The pooled prevalence of TBEV in cheese made from raw milk was 3% (95% CI 0%–13%; Figure 4, Table 2).

### 3.3 Critical Appraisal of Included Studies

Of the six studies assessed for methodological quality using the prevalence tool, no studies had a high quality, two studies had a moderate quality, and the remaining studies had low quality.

Of the 10 studies (outbreak investigations) assessed using the case series tool, three studies scored as high quality, and the remaining studies were given a low rating. Details of the critical appraisal evaluation are included in Data S6.

## 4 Discussion

This systematic review affirms that raw milk and its derivatives from goats, sheep and cows can potentially harbour TBEV. It helped to estimate the occurrence or prevalence of TBE virus/antibodies in dairy products, highlighting the potential infection risk for consumers.

Most of the available data refer to Austria, Croatia, Slovakia and Sweden. It is important to note that there is a significant lack of published research on TBEV transmission to humans through milk and dairy products in the EU countries. This gap in research could be attributed to different reasons. First, research in Europe has been focusing on the main, vector‐borne, transmission route. Secondly, research might be restricted to a few countries, where the virus has high circulation levels and where the consumption of raw milk products is more common. EU Member States can restrict the sale of unpasteurised milk and milk products intended for human consumption according to food hygiene rules (EFSA 2015). However, the consumption of such products is very popular in some areas, linked to different reasons, including health reasons (e.g., the perception that these products are a ‘healthier’ choice) and organoleptic qualities, but also as a means to support local farmers (Berge and Baars 2020).

Most of the studies included in this review are focused on goat products. Notably, human outbreaks in Europe have often been traced back to the consumption of raw goat milk and cheese (Martello et al. 2022). This raises critical questions regarding the duration and intensity of viremia in goats, as also discussed by Markovinović and colleagues (Markovinović et al. 2016). Alimentary human cases originating from the consumption of sheep and cow raw milk and products are reported with a lower extent (Martello et al. 2022). Besides this, our systematic review shows a variable prevalence of TBEV in milk and products (0%–28%) in accordance with the variable prevalence when testing ticks as reported in the literature (0.1%–37.3%) (Imhoff et al. 2015; Bormane et al. 2004). Thus, milk products testing results also seem to reflect the focal nature of TBEV.

Most of the studies were conducted in the aftermath of outbreak investigations (*n* = 10) and the others were designed as prevalence studies (*n* = 6). It is worth noting that outbreak investigations could introduce bias into prevalence estimates, as they inherently increase the likelihood of detecting positive samples, and they often target suspected animals as the source of infection. Nevertheless, this bias is not universal, as evidenced by most of our outbreak investigation studies, where a wide range of prevalence was estimated, including studies where none of the tested samples resulted positive to TBEV (Markovinović et al. 2016; Holzmann et al. 2009; Caini et al. 2012; Brockmann et al. 2018; Ilic et al. 2020; Paraličová et al. 2022). Indeed, the time interval between the detection of human cases and animal sampling is crucial in the identification of positive samples, as the viral excretion time varies among animals' species and single individuals (Ličková et al. 2022). Moreover, in outbreak investigations, the possible source of infection may not be available for testing, e.g. when there are not leftovers of the original potentially infected cheese (Holzmann et al. 2009; Paraličová et al. 2022).

Prevalence studies were mainly conducted in countries/study areas where an increasing incidence of human TBE cases has been reported. Three studies took place in farms located across a whole country, while farms from small areas at high risk or with recent/emerging TBE risk within a country are the object of the other three studies. Authors propose testing milk as a way of mapping prevalence across the territory and detecting new TBEV foci (Blomqvist et al. 2021; Pautienius et al. 2021).

The results interpretation for the different types of data collected in our included papers should be carefully considered. While similar prevalence estimates were found for goat and cow (3%–4%) when considering individual milk samples, the only study on sheep reported a prevalence of 22%. This result is difficult to generalise; however, the study was performed in an area with a high risk of TBEV infection and also reports a high infection prevalence in goat and cow milk samples (20.7% and 11.1%, respectively). The use of single animal data, including testing individual milk, can be useful for getting relevant information about the infection dynamics. Moreover, the sensitivity of the test is higher compared to testing tank milk when only a few individuals are infected. However, individual testing could be less useful due to the irregular distribution and patchy geographical occurrence of TBEV, which can make it difficult to detect the virus in some areas and has higher costs compared to bulk milk testing.

In this review, the prevalence estimates for TBEV in bulk milk were too heterogeneous to conduct a meta‐analysis. Evaluating bulk tank milk from a herd requires careful consideration, and even within herds classified as seronegative based on bulk tank milk samples, individual positive samples can still be present (Blomqvist et al. 2021). Consequently, using tank milk samples for sentinel monitoring has some limitations in terms of sensitivity (Cisak et al. 2010). However, the use of bulk milk samples offers significant advantages: it is an easy and cost‐effective way to monitor for infections because one sample can represent several individuals, who might have been exposed to the disease. It is especially useful when taken at the end of the grazing season or vector period to maximise the chances of detecting infection. Additionally, integrating the mapping of TBEV presence into existing national control programs using tank milk samples collected for any bovine disease surveillance is a practical approach (Blomqvist et al. 2021).

The pooled prevalence of TBEV in cheese made from goat or mixed goat and cow raw milk was very similar to the results on bulk milk (3%). TBEV was detected in ‘fresh’ cheese, with the exception of one study, where the virus was detected in one out of two samples of ripened cheese. In the same study (Brockmann et al. 2018), the viability of TBEV in goat cream cheese was demonstrated by cell culture. In two studies, tested cheese was negative by PCR; in one case, it was fresh cheese; in the other study, no details on the cheese type are given. The source of TBEV infection is thus generally fresh cheese, sold directly by farmers at the farm soon after production. However, according to the literature, TBEV can survive up to 15 days in unsalted goat cheese samples, with or without spices; apart from milk pasteurisation, salt treatment seems critical to destroy infective TBEV particles in cheese (Rónai and Egyed 2020).

In our review, we observed that researchers exhibited variability in their choice of direct or indirect assays for testing sample positivity. Consequently, we opted to conduct an analysis that segregated this data, giving preference to PCR data when multiple detection methods were employed. To date, even if the detection of viral genomes cannot be correlated to the presence of infectious viruses, PCR assays are used to carry out prevalence studies in food matrices due to their high sensitivity and to the fact that they can be employed when reliable cell‐culture methods are not available.

### 4.1 Strengths and Limitations of the Systematic Review

This systematic review has several significant strengths, particularly its strict adherence to the JBI methodology and the PRISMA guidelines throughout the entire process. All the studies underwent independent double screening of titles, abstracts and full texts, ensuring a rigorous approach. These meticulous methodological practices have effectively minimised the possibility of excluding relevant studies, ultimately bolstering the validity and reliability of the systematic review.

However, there are some limitations. Although an exhaustive systematic search was conducted, for each country our findings are based on few studies. Furthermore, despite our comprehensive searches and inclusive screening processes without language restrictions, we were able to identify eligible studies from only 12 countries. Including grey literature would have been beneficial. The included studies varied by study design (outbreak investigation and prevalence study) reflecting the different underlying purposes, which could have affected the results of the meta‐analysis, though introducing heterogeneity between the studies. Unfortunately, we were not able to conduct a subgroup analysis to assess whether there were significant differences by study design due to the low number of studies.

The methodological quality of the included studies varied, with three studies being deemed high quality, two moderate and the rest low. Although studies reported their methods well in terms of the sample analysis and identification of TBEV, the lower quality of some of the included studies could have impacted the findings of our meta‐analysis by introducing additional uncertainty in the estimated pooled result. However, meta‐analysis can still be useful to identify whether findings from studies are consistent, sources of disagreement, and whether patterns exist across studies. We recommended future studies provide a fuller description of sampling selection, sample characteristics and the reporting of the main settings of the study in a more consistent way (including farming systems).

## 5 Conclusions and Recommendations

According to our review, only limited efforts have been made to evaluate safety in milk and milk products in terms of TBEV infection. There is an increasing awareness of the occurrence of alimentary outbreaks (Martello et al. 2022), indicative of the possibility of acquiring TBEV infection via alimentary transmission, and not only following a tick bite. Therefore, we need to better understand what type of farms are at higher risk (type of production e.g., animal in/out, animal breeds), what cheese type is at risk (e.g., what is the survival of the virus in the cheese), what is the infectious dose, and if human vaccination would protect against infection via the alimentary route. This proactive approach can serve as an additional data source, especially when coupled with public education initiatives highlighting the potential risks associated with untreated milk products. Concurrently, it is relevant to continue gathering data on tick vectors and animal hosts to comprehensively understand the dynamics of TBEV transmission in different geographic areas.

The ease of obtaining dairy products and conducting tests on them can greatly aid in risk assessment and contribute to the broader epidemiological surveillance of TBE within a One Health framework. Periodic milk testing could integrate other data collection systems, such as the European Surveillance System (‘TESSy’) of the European Centre for Disease Prevention and Control (ECDC), which will start collecting information on the mode of TBEV transmission, not only in the case of outbreaks. However, it is likely that TBEV alimentary transmission will be mostly recorded during outbreak investigations characterised by cases with a strong epidemiological link.

Finally, immunisation of goats could be suggested in endemic areas and where the consumption of raw milk products is high, as an effective method of preventing TBEV infection via the alimentary route (Balogh et al. 2012).

## Author Contributions

E.M. and L.T. were the lead reviewers. J.L.‐B. performed the analysis. All authors contributed to the drafting of the manuscript, interpretation and communication of the results.

## Conflicts of Interest

The authors declare no conflicts of interest.

## Supporting information


**Data S1.** Protocol.

**Data S2.** Countries eligible for inclusion in this systematic review.

**Data S3.** Search strategy for the three electronic databases used.

**Data S4.** List of excluded studies with reason.

**Data S5.** Diagnostic tests used in the 16 included papers.

**Data S6.** Critical appraisal of included studies.

## Data Availability

Data that support the findings of this study are available from the corresponding author upon request.
