# Supplementary material for: A Systematic Review on the Prevalence of Tick‐Borne Encephalitis Virus in Milk and Milk Products in Europe
Source: Zoonoses Public Health. 2025 Feb 23;72(3):248–58. doi: 10.1111/zph.13216 (PMC11967290; doi:10.1111/zph.13216)
Supplement: Supplementary file 1 — Data S1. Protocol. [file ZPH-72-248-s005.pdf]

Tick-borne encephalitis infection in milk and dairy products from domestic ruminants in Europe: a systematic literature review

Citation

Alessandro Mannelli, Aurora Vicentini, Elisa Martello, Jo Leonardi-Bee, Laura Tomassone. Tick-borne encephalitis infection in milk and dairy products from domestic ruminants in Europe: a systematic literature review. PROSPERO 2021 CRD42021279317 Available from:
https://www.crd.york.ac.uk/prospero/display_record.php?ID=CRD42021279317

Review question

To estimate the prevalence of TBEV in raw milk, cheese and other dairy products from cow, sheep, and goats in Europe

Context and rationale

Tick-borne encephalitis virus (TBEV) is a Flavivirus responsible for one of the most important zoonosis, with thousands of human cases per year in Europe. Although TBE represents a vector-borne disease and the main human infection route is the bite of an infected tick, humans can also be infected through the consumption of raw milk from viraemic ruminants (cow, sheep, and goats) and their cheese made with raw milk. Furthermore, raw milk represents an effective method to identify TBEV presence in a geographical area, as it allows for a wider number of animals to be monitored, compared to individual sampling.Considering the absence of a systematic review which summarises TBEV prevalence in raw milk, cheese and other dairy products from domestic ruminants, a comprehensive overview is needed to quantify the relevance of the alimentary route of transmission and to provide important information for human TBE risk assessments.

Searches [1 change]

A comprehensive search using three electronic databases (MEDLINE, EMBASE and CAB Abstracts; OVID platform) will be used to identify peer-reviewed articles, which assess the prevalence of TBEV in raw milk, cheese and other dairy products. We will use and appropriately combine the following search terms, using subject headings and freetext words: Tick-borne encephalitis, Tick borne encephalitis virus, TBE, TBEV, Tick borne encephalit*, Tick-borne encephalit*, Tickborne encephalit*, goat, cow, cattle, sheep, milk, cheese, milk product*, dairy, yogurt. We will limit our search to studies published between 1980 to present. We will check the reference list of the included studies. No further studies will be identified through hand searching of grey literature sources and we will not consider unpublished literature.

Study designs to be included

Inclusion criteria:

Cross-sectional and cohort/longitudinal designed observational studies; descriptive studies (case reports and case series)

Exclusion criteria:

Any other study design different from observational and descriptive studies

Human disease modelled

Tick-borne encephalitis virus (TBEV) is responsible for one of the most important zoonosis, with thousands of human cases per year in Europe and, in the last few decades, an increasing presence and a wider distribution of TBEV have been registered both in latitude and altitude. While most cases are asymptomatic, it may cause meningitis or meningoencephalitis and the mortality rate is about 2% but long-lasting sequelae are observed in 50% of adult patients.

Animals/population

Inclusion criteria:

Raw milk and derived products from three animal species: cow, sheep, and goat

Exclusion criteria:

Any other animal species and any other substratum

Intervention(s), exposure(s) [1 change]

Inclusion criteria:

None

Exclusion criteria:

None

Comparator(s)/control [1 change]

Inclusion criteria:

None

Exclusion criteria:

None

Other selection criteria or limitations applied [1 change]

We will include only studies with title and abstract in English and available full text in English, German, French, Spanish or Italian. We will limit our search to studies conducted in Europe. Therefore, we will exclude publications without title or abstract and full text not available.

Outcome measure(s)

Inclusion criteria:

The primary outcome is TBEV test positivity. We will consider studies which performed any laboratory test on raw milk or cheese or other dairy products (e.g., yogurt) to identify TBEV or Ab anti-TBEV and which provide numerical data (e.g., number of tested animal or tested sample of milk/cheese, number of positive animals or samples).

Exclusion criteria:

No numerical data reported about TBEV test positivity in raw milk or cheese or other dairy products from cow, sheep, and goats in Europe

Study selection and data extraction [1 change]

Procedure for study selection

We will pilot title and abstract screening for 500 hits by two reviewers independently, any discrepancies will be resolved discussing with a third reviewer. If a high level of agreement (>90%) is reached, the remaining titles and abstracts can be

screened by one reviewer; or a further round of piloting will be performed.

Prioritise the exclusion criteria

Title-abstract screening:

1. Not raw milk and derived products from cow, sheep, and goat

2. Studies not conducted in Europe

3. Studies conducted before 1980

4. Not cross-sectional and cohort/longitudinal designed observational studies or descriptive studies

Full text-screening:

as above, with the addition of:

5. No numerical data reported

Methods for data extraction

Data extraction will be conducted by two reviewers independently; any disagreement will be discussed with a third reviewer.

Data to be extracted: study design

Type of sampling, number of animal or sample tested, stratification criteria and clustering factors.

Data to be extracted: animal model

Animal species, sample tested, characteristics of the farm

Data to be extracted: intervention of interest

None

Data to be extracted: primary outcome(s)

Number of positive animals or samples; continuous. Prevalence of positive samples will be calculated by using number of positive divided by the number of tested samples.

Data to be extracted: secondary outcome(s)

None

Data to be extracted: other

Author name and year of publication; diagnostic test and date of diagnosis, Country, specific location; TBE prevalence in ticks and human cases in the same area.

Risk of bias and/or quality assessment

Other criteria, namely JBI Critical Appraisal tools for Prevalence and Incidence Studies, Case Reports and Case Series.

Quality of each included study will be assessed using the JBI Critical Appraisal tools for Prevalence and Incidence Studies, Case Reports and Case Series by two reviewers independently..

Strategy for data synthesis [1 change]

Planned approach

We anticipated a high level of heterogeneity between the studies due to differences in characteristics and methodologies of the included studies; therefore, where possible we will estimate a pooled prevalence, with 95% confidence intervals, using a random effects model. The feasibility of conducting a meta-analysis will depend on the comparability of the studies included in the systematic review based on the study design and animal species considered. The decision for conducting a meta-analysis will not be based on a threshold for heterogeneity since we expect high levels of heterogeneity between studies, nor a minimal number of studies.

Effect measure

Where possible we will estimate a pooled prevalence, with 95% confidence intervals,

Effect models

Random effects model

Heterogeneity

Heterogeneity will be quantified using I^2

Other

None

Analysis of subgroups or subsets [1 change]

Subgroup analyses

Where data permit, we will perform sub-group analysis animal species, sample, country, and method of diagnosis

Sensitivity

The certainty of evidence will be ranked using GRADE, where applicable.

Publication bias

None

Contact details for further information

Alessandro Mannelli

alessandro.mannelli@unito.it

Organisational affiliation of the review

Department of Veterinary Sciences, University of Turin

https://www.veterinaria.unito.it/do/home.pl

Review team members and their organisational affiliations

Professor Alessandro Mannelli. Department of Veterinary Sciences, University of Turin

Miss Aurora Vicentini. Department of Veterinary Sciences, University of Turin

Dr Elisa Martello. Centre for Evidence Based Healthcare, Division of Epidemiology and Public Health, University of Nottingham

Professor Jo Leonardi-Bee. Centre for Evidence Based Healthcare, Division of Epidemiology and Public Health, University of Nottingham

Professor Laura Tomassone. Department of Veterinary Sciences, University of Turin

Review type

Animal model review

Anticipated or actual start date

15 July 2021

Anticipated completion date

15 January 2022

Funding sources/sponsors

Department of Veterinary Sciences, University of Turin, Turin, Italy

Conflicts of interest

Language

English

Country

England, Italy

Stage of review

Review Ongoing

Subject index terms status

Subject indexing assigned by CRD

Subject index terms

Animals; Encephalitis, Tick-Borne; Europe; Milk; Ruminants; Tick-Borne Diseases

Date of registration in PROSPERO

29 October 2021

Date of first submission

28 September 2021

Stage of review at time of this submission

Preliminary searches	Yes	Yes
Piloting of the study selection process	No	No
Formal screening of search results against eligibility criteria	No	No
Data extraction	No	No
Risk of bias (quality) assessment	No	No
Data analysis	No	No

The record owner confirms that the information they have supplied for this submission is accurate and complete and they understand that deliberate provision of inaccurate information or omission of data may be construed as scientific misconduct.

The record owner confirms that they will update the status of the review when it is completed and will add publication details in due course.

Versions

29 October 2021
