# Supplementary material for: A Systematic Review on the Prevalence of Tick‐Borne Encephalitis Virus in Milk and Milk Products in Europe
Source: Zoonoses Public Health. 2025 Feb 23;72(3):248–58. doi: 10.1111/zph.13216 (PMC11967290; doi:10.1111/zph.13216)
Supplement: Supplementary file 2 — Data S2. Countries eligible for inclusion in this systematic review. [file ZPH-72-248-s001.docx]

# Supplement S2. Countries eligible for inclusion in this systematic review

The following countries were eligible for inclusion in the systematic review:

1. Austria
2. Belgium
3. Bulgaria
4. Croatia
5. Cyprus
6. Czechia
7. Denmark
8. Estonia
9. Finland
10. France
11. Germany
12. Greece
13. Hungary
14. Iceland
15. Ireland
16. Italy
17. Latvia
18. Lithuania
19. Luxembourg
20. Malta
21. Netherlands
22. Norway
23. Poland
24. Portugal
25. Romania
26. Slovakia
27. Slovenia
28. Spain
29. Sweden
30. Switzerland
31. UK
