# Supplementary material for: A Systematic Review on the Prevalence of Tick‐Borne Encephalitis Virus in Milk and Milk Products in Europe
Source: Zoonoses Public Health. 2025 Feb 23;72(3):248–58. doi: 10.1111/zph.13216 (PMC11967290; doi:10.1111/zph.13216)
Supplement: Supplementary file 3 — Data S3. Search strategy for the three electronic databases used. [file ZPH-72-248-s002.docx]

**Supplement S3**

Search strategy for the three electronic databases used:

**Embase <1980 to 2022 Week 43>**

1 Tick-borne encephalitis/

2 Tick borne encephalitis virus/

3 TBEv.mp.

4 TBE.mp.

5 Tick borne encephalit*.mp.

6 Tick-borne encephalit*.mp.

7 Tickborne encephalit*.mp. [mp=title, abstract, heading word, drug trade name, original title, device manufacturer, drug manufacturer, device trade name, keyword heading word, floating subheading word, candidate term word]

8 1 or 2 or 3 or 4 or 5 or 6 or 7

9 8 and (goat or cow or cattle or sheep).mp. [mp=title, abstract, heading word, drug trade name, original title, device manufacturer, drug manufacturer, device trade name, keyword heading word, floating subheading word, candidate term word]

10 9 and (milk or milk product* or cheese or dairy or yogurt).mp. [mp=title, abstract, heading word, drug trade name, original title, device manufacturer, drug manufacturer, device trade name, keyword heading word, floating subheading word, candidate term word]

11 8 and (9 or 10)

12 limit 11 to yr="1980 -Current"

**CAB Abstracts <1973 to 2022 Week 43>**

1 Tick-borne encephalitis/

2 Tick borne encephalitis virus/

3 TBEv.mp.

4 TBE.mp.

5 Tick borne encephalit*.mp.

6 Tick-borne encephalit*.mp.

7 Tickborne encephalit*.mp. [mp=abstract, title, original title, broad terms, heading words, identifiers, cabicodes]

8 1 or 2 or 3 or 4 or 5 or 6 or 7

9 8 and (goat or cow or cattle or sheep).mp. [mp=abstract, title, original title, broad terms, heading words, identifiers, cabicodes]

10 9 and (milk or milk product* or cheese or dairy or yogurt).mp. [mp=abstract, title, original title, broad terms, heading words, identifiers, cabicodes]

11 8 and (9 or 10)

12 limit 11 to yr="1980 -Current"

**Ovid MEDLINE(R) ALL <1946 to October 31, 2022>**

1 Tick-borne encephalitis/

2 Tick borne encephalitis virus/

3 TBEv.mp.

4 TBE.mp.

5 Tick borne encephalit*.mp.

6 Tick-borne encephalit*.mp.

7 Tickborne encephalit*.mp. [mp=title, book title, abstract, original title, name of substance word, subject heading word, floating sub-heading word, keyword heading word, organism supplementary concept word, protocol supplementary concept word, rare disease supplementary concept word, unique identifier, synonyms]

8 1 or 2 or 3 or 4 or 5 or 6 or 7

9 8 and (goat or cow or cattle or sheep).mp. [mp=title, book title, abstract, original title, name of substance word, subject heading word, floating sub-heading word, keyword heading word, organism supplementary concept word, protocol supplementary concept word, rare disease supplementary concept word, unique identifier, synonyms]

10 9 and (milk or milk product* or cheese or dairy or yogurt).mp. [mp=title, book title, abstract, original title, name of substance word, subject heading word, floating sub-heading word, keyword heading word, organism supplementary concept word, protocol supplementary concept word, rare disease supplementary concept word, unique identifier, synonyms]

11 8 and (9 or 10)

12 limit 11 to yr="1980 -Current"
