# Supplementary material for: A Systematic Review on the Prevalence of Tick‐Borne Encephalitis Virus in Milk and Milk Products in Europe
Source: Zoonoses Public Health. 2025 Feb 23;72(3):248–58. doi: 10.1111/zph.13216 (PMC11967290; doi:10.1111/zph.13216)
Supplement: Supplementary file 4 — Data S4. List of excluded studies with reason. [file ZPH-72-248-s003.docx]

**Supplement S4. List of excluded studies with reason.**

| **First Author(s)** | **Title** | **Year of pubblication** | **Reason for exclusion** |
| --- | --- | --- | --- |
| Van den Brom et al. (1) | Zoonotic risks of pathogens from sheep and their milk borne transmission | 2020 | ineligible study design |
| EFSA (2) | Scientific opinion on the public health risks related to the consumption of raw drinking milk | 2015 |  |
| Labuda et al. (3) | Tick-borne encephalitis virus foci in Slovakia. | 2001 |  |
| Knezevic et al. (4) | Tick-borne encephalitis due to consumption of raw goat milk, gorski kotar, 2019: Clinical case reports | 2019 | abstract only |
| Ingenhoff et al. (5) | Risk assessment of the alimentary transmission of tick-borne encephalitis viruses from goats to humans by milk and milk products in Swiss alpine regions | 2020 |  |
| Kontrosova and Strharsky (6) | Tick-borne encephalitis - an epidemiological study in the Slovak Republic. | 2002 |  |
| Zaludko et al. (7) | Familial epidemics of tick-borne encephalitis in central Povazie | 1994 | ineligible participants (non-domestic ruminants)/substratum (not milk or milk products) |
| Klaus et al. (8) | Tick-borne encephalitis virus (TBEV) antibodies in animal sera - Occurrence in goat flocks in Germany, longevity and ability to recall immunological information after more than six years | 2019 |  |
| Dorko et al. (9) | Milk outbreaks of tick-borne encephalitis in Slovakia, 2012-2016 | 2018 |  |
| Kaiser (10) | Tick-borne encephalitis | 2016 |  |
| Roelandt et al. (11) | Autochthonous tick-borne encephalitis virus (TBEV)-seropositive cattle in Belgium: A risk-based targeted serological survey | 2014 |  |
| Kerbo et al. (12) | Tickborne encephalitis outbreak in Estonia linked to raw goat milk, May-June 2005 | 2005 |  |
| Zeman et al. (13) | High seroprevalence of granulocytic ehrlichiosis distinguishes sheep that were the source of an alimentary epidemic of tick-borne encephalitis | 2004 |  |
| Rieger et al. (14) | Tick-borne encephalitis transmitted by raw milk--what is the significance of this route of infection? Studies in the epidemic region of South-West Germany | 1998 |  |
| Aendekerk et al. (15) | Tick-borne encephalitis complicated by a polio-like syndrome following a holiday in central Europe | 1996 |  |
| Chitimia-Dobler et al. (16) | Tick-borne encephalitis vaccination protects from alimentary TBE infection: results from an alimentary outbreak | 2021 |  |
| Krol et al. (17) | Outbreak of alimentary tick-borne encephalitis in Podlaskie voivodeship, Poland | 2019 |  |
| Kerlik et al. (18) | Slovakia reports highest occurrence of alimentary tick-borne encephalitis in Europe: analysis of tick-borne encephalitis outbreaks in Slovakia during 2007-2016 | 2018 |  |
| Salat et al. (19) | Tick-borne encephalitis in small ruminants | 2017 |  |
| Verraes et al. (20) | A review of the microbiological hazards of raw milk from animal species other than cows. | 2014 |  |
| Zoldi et al. (21) | Milk transmitted tick-borne encephalitis epidemics in Hungary. | 2013 |  |
| Balogh et al. (22) | Tick-borne encephalitis outbreak in Hungary due to consumption of raw goat milk. | 2010 |  |
| Kriz et al. (23) | Alimentary transmission of tick-borne encephalitis in the Czech Republic (1997-2008). | 2009 |  |
| Nekrosiene et al. (24) | Seroepizootic survey of tick-borne encephalitis in goats in Lithuania. | 2003 |  |
| Botiakow et al. (25) | Tick-borne western encephalitis in the region of the Bialowieza forest. | 1986 |  |
| Salat et al. (26) | Sero-epidemiology of tick-borne encephalitis in small ruminants in the Czech Republic. | 2022 |  |
| Matuszczyk et al. (27) | The outbreak of an epidemic of tick-borne encephalitis in Kielec province induced by milk ingestion | 1997 | duplicate |
| Ferenczi et al. (28) | Tick-borne encephalitis outbreaks through raw milk consumption in Hungary. | 2009 |  |
| Matuszczyk et al. (29) | An outbreak of milky epidemic of encephalitis caused by tick-borne encephalitis virus in Kielce Province. | 1997 |  |
| NA (30) | Outbreak of tick-borne encephalitis, presumably milk-borne. Slovakia. | 1994 |  |
| Sixl et al. (31) | Rare transmission mode of FSME (tick-borne encephalitis) by goat's milk | 1989 | review |
| Bojanić Rasovic (32) | The importance of transmission tick-borne encephalitis through milk of infected animals | 2018 |  |
| Klaus et al. (33) | Tick-borne encephalitis virus infections in animals - clinical symptoms, diagnostics and epidemiologic relevance | 2017 |  |
| Laczay et al. (34) | Public health significance of milk-borne pathogens | 2016 |  |
| Salat and Ruzek (35) | Tick-borne encephalitis in domestic animals | 2020 |  |
| Kohl et al. (36) | Studies of a natural focus of tick-borne encephalitis in the Povazska Bystrica district. | 1989 | unclear content |

1. van den Brom R, de Jong A, van Engelen E, Heuvelink A, Vellema P. Zoonotic risks of pathogens from sheep and their milk borne transmission. Small Rumin Res. 2020;189:106123. doi: 10.1016/j.smallrumres.2020.106123

2. EFSA. (2015). BIOHAZ. Scientific Opinion on the public health risks related to the consumption of raw drinking milk. EFSA Journal. 13. 10.2903/j.efsa.2015.3940.

3. Labuda M, Elečková E, Ličková M, Sabó A. Tick-borne encephalitis virus foci in Slovakia. International Journal of Medical Microbiology, 291 (33), 2002 : 43-47. https://doi.org/10.1016/S1438-4221(02)80008-X.

4. Knežević S, Slavuljica I, Lakošeljac D, Vicković N, Cekinović Grbeša D, Gorup L, Bubonja Šonje M, Rončević D, Ilić M, Bogdanić M. Tick-Borne Encephalitis Due to Consumption of Raw Goat Milk, Gorski kotar, 2019: Clinical Case Reports. INFEKTOL GLASN 2019;39(3):93-97. <https://doi.org/10.37797/ig.39.3.4>

5. Ingenhoff J.-E., Mühlemann M., Ackermann-Gäumann R., Moor D., Berger T. Risk assessment of alimentary transmission of tick-borne encephalitis viruses from goats to humans by means of milk and milk products in alpine regions of Switzerland. nIn: IDF International Cheese Science and Technology Symposium, June 7, 9 and 11, 2021. 07.06., nHrsg. IDF, IDF, virtual (Quebec). 2021.

6. Kontrosova S., Strharsky J. Tick-borne encephalitis - an epidemiological study in the Slovak Republic. Kliesthacekova encefalitida - epidemiologicka situacia v sr. 2002, Volume 11, Issue 6, pp. 37-38

7. Zaludko J, Vrbová O, Hachlincová R, Kohl I, Hubálek Z, Jurincová Z, Kozuch O, Elecková E, Labuda M. Rodinné epidémie kliest'ovej encefalitídy na strednom Povazí [Familial epidemics of tick-borne encephalitis in central Povazie]. Bratisl Lek Listy. 1994 Nov;95(11):523-6.

8. Klaus C, Ziegler U, Hoffmann D, Press F, Fast C, Beer M. Tick-borne encephalitis virus (TBEV) antibodies in animal sera - occurrence in goat flocks in Germany, longevity and ability to recall immunological information after more than six years. BMC Vet Res. 2019 Nov 6;15(1):399. doi: 10.1186/s12917-019-2157-5.

9. Dorko E, Hockicko J, Rimárová K, Bušová A, Popaďák P, Popaďáková J, Schréter I. Milk outbreaks of tick-borne encephalitis in Slovakia, 2012-2016. Cent Eur J Public Health. 2018 Dec;26 Suppl:S47-S50. doi: 10.21101/cejph.a5272.

10. Kaiser R. Frühsommermeningoenzephalitis [Tick-borne encephalitis]. Nervenarzt. 2016 Jun;87(6):667-80. German. doi: 10.1007/s00115-016-0134-9. PMID: 27225401.

11. Roelandt S, Suin V, Riocreux F, Lamoral S, Van der Heyden S, Van der Stede Y, Lambrecht B, Caij B, Brochier B, Roels S, Van Gucht S. Autochthonous tick-borne encephalitis virus-seropositive cattle in Belgium: a risk-based targeted serological survey. Vector Borne Zoonotic Dis. 2014 Sep;14(9):640-7. doi: 10.1089/vbz.2014.1576.

12. Kerbo N, Donchenko I, Kutsar K, Vasilenko V. Tickborne encephalitis outbreak in Estonia linked to raw goat milk, May-June 2005. Euro Surveill. 2005 Jun 23;10(6):E050623.2. doi: 10.2807/esw.10.25.02730-en.

13. Zeman, P., Januska, J., Orolinova, M. et al. High seroprevalence of granulocytic ehrlichiosis distinguishes sheep that were the source of an alimentary epidemic of tick-borne encephalitis. Wien Klin Wochenschr 116, 614–616 (2004). <https://doi.org/10.1007/s00508-004-0191-0>

14. Rieger MA, Nübling M, Kaiser R, Tiller FW, Hofmann F. Tick-borne encephalitis transmitted by raw milk--what is the significance of this route of infection? Studies in the epidemic region of South-West Germany. Gesundheitswesen. 1998 Jun;60(6):348-56.

15. Aendekerk RP, Schrivers AN, Koehler PJ. Tick-borne encephalitis complicated by a polio-like syndrome following a holiday in central Europe. Clin Neurol Neurosurg. 1996 Aug;98(3):262-4. doi: 10.1016/0303-8467(96)00030-3

16. Chitimia-Dobler L, Lindau A, Oehme R, Bestehorn-Willmann M, Antwerpen M, Drehmann M, Hierl T, Mackenstedt U, Dobler G. Tick-Borne Encephalitis Vaccination Protects from Alimentary TBE Infection: Results from an Alimentary Outbreak. Microorganisms. 2021 Apr 21;9(5):889. doi: 10.3390/microorganisms9050889.

17. Monika Emilia Król, Bartłomiej Borawski, Anna Nowicka-Ciełuszecka, Jadwiga Tarasiuk, Joanna Zajkowska. Outbreak of alimentary tick-borne encephalitis in Podlaskie voivodeship, Poland. Przegl Epidemiol. 2019;73(2):239-248. doi: 10.32394/pe.73.01.

18. Kerlik J, Avdičová M, Štefkovičová M, Tarkovská V, Pántiková Valachová M, Molčányi T, Mezencev R. Slovakia reports highest occurrence of alimentary tick-borne encephalitis in Europe: Analysis of tick-borne encephalitis outbreaks in Slovakia during 2007-2016. Travel Med Infect Dis. 2018 Nov-Dec;26:37-42. doi: 10.1016/j.tmaid.2018.07.001.

19. Salat J, Slosarkova, S.; Ruzek, D. Tick-borne encephalitis in small ruminants. Klist'ova encefalitida u malych prezvykavcu. 2017, Volume 67, Issue 8, pp. 627-631

20. Verraes C., W. Claeys, S. Cardoen, G. Daube, L. De Zutter, H. Imberechts, K. Dierick, L. Herman. A review of the microbiological hazards of raw milk from animal species other than cows. International Dairy Journal, Volume 39, Issue 1, 2014, Pages 121-130, ISSN 0958-6946, <https://doi.org/10.1016/j.idairyj.2014.05.010>.

21. Zöldi V, Ferenczi E, Egyed L. Milk-transmitted tick-borne encephalitis epidemics in Hungary. Magyar Állatorvosok Lapja. 2013;135:48-56.

22. Balogh Z, Ferenczi E, Szeles K, Stefanoff P, Gut W, Szomor KN, Takacs M, Berencsi G. Tick-borne encephalitis outbreak in Hungary due to consumption of raw goat milk. J Virol Methods. 2010 Feb;163(2):481-5. doi: 10.1016/j.jviromet.2009.10.003.

23. Kríz B, Benes C, Daniel M. Alimentary transmission of tick-borne encephalitis in the Czech Republic (1997-2008). Epidemiol Mikrobiol Imunol. 2009 Apr;58(2):98-103.

24. Nekrošienė N, Bagdonas J, Žygutienė M. Seroepizootic survey of tick-borne encephalitis in goats in Lithuania. Lietuvos mokslų akademija 2003, p. 58-63

25. Botiakow W, Samojłowa T, Protas I. Western tick-borne encephalitis in the region of the Białowieza Forest. Przeglad Epidemiologiczny 1986, p. 188-192

26. Salat J, Strakova P, Stefanik M, Slosarkova S, Ruzek D. Sero-epidemiology of tick-borne encephalitis in small ruminants in the Czech Republic. Ticks Tick Borne Dis. 2022 Sep;13(5):101996. doi: 10.1016/j.ttbdis.2022.101996.

27. Matuszczyk I, Tarnowska H, Zabicka J, Gut W. The outbreak of an epidemic of tick-borne encephalitis in Kielec province induced by milk ingestion. Przegl Epidemiol. 1997;51(4):381-8.

28. Ferenczi, E ; Széles, K ; Balogh, ZS ; Gut, W. Tick-borne encephalitis outbreaks through raw milk consumption in Hungary. In: Benkő, M; Harrach, B (eds.) 8th International Congress of Veterinary Virology : 20 years of ESVV: Integrating classical and molecular virology; Programme and Proceedings Bp, Hungary : MTA Állatorvos-tudományi Kutatóintézet (2009) 248 p., p. 123

29. Matuszczyk I, Tarnowska H, Zabicka J, Gut W, 1997. An outbreak of milky epidemic of encephalitis caused by tick-borne encephalitis virus in Kielce Province. Przeglad Epidemiologiczny, 51(4):381-388

30. NA. Outbreak of tick-borne encephalitis, presumably milk-borne. Wkly Epidemiol Rec. 1994 May 13;69(19):140-1. English, French. PMID: 8025013.

31. Sixl W, Stünzner D, Withalm H, Köck M. Rare transmission mode of FSME (tick-borne encephalitis) by goat's milk. Geogr Med Suppl. 1989;2:11-4.

32. Bojanić Rašović M. The importance of transmission tick-borne encephalitis through milk of infected animals. Journal of Hygienic Engineering and Design 25, 2018.

33. Klaus C., Hoffmann D., Hoffmann B., Beer M. Tick-borne encephalitis virus infections in animals – clinical symptoms, diagnostics and epidemiologic relevance. Berl Münch Tierärztl Wochenschr, DOI 10.2376/0005-9366-16062

34. Laczay P., Lehel J, Lanyl K, Laszlo N. Public health significance of milk-borne pathogens. Magyar Allatorvosok lapja, 2016, 138: 231-242

35. Salat J, Ruzek D. Tick-borne encephalitis in domestic animals. Acta Virol. 2020;64(2):226-232. doi: 10.4149/av_2020_212.

36. Kohl I, M. Gresikova, V. Kohutova, J. Zaludko, M. Sekeyova. Studies of a natural focus of tick-borne encephalitis in the Povazska Bystrica district. Vysk. Prir. Ohniska Kliesthacek∼Ovej Encef. (KE) v Okr. Povazska Bystrica, 44 (1989), pp. 267-273
