# Supplementary material for: A Systematic Review on the Prevalence of Tick‐Borne Encephalitis Virus in Milk and Milk Products in Europe
Source: Zoonoses Public Health. 2025 Feb 23;72(3):248–58. doi: 10.1111/zph.13216 (PMC11967290; doi:10.1111/zph.13216)
Supplement: Supplementary file 5 — Data S5. Diagnostic tests used in the 16 included papers. [file ZPH-72-248-s006.docx]

| **Paper** | **Test** |
| --- | --- |
| Kohl06 | Virus neutralization test (detailed protocol) |
| Holzmann2009 | PCR (no details, no references to published protocols) |
| Cisak2010 | Immunozym FSME IgG All Species (Progen Biotechnik GMBH, Heidelberg, Germany)  AND  Nested RT-PCR (own protocol described in detail) |
| Caini2012 | PCR (no details, no references to published protocols) |
| Hudopisk2013 | Indirect immunofluorescent assay (no details reported)  AND  RT-PCR (reference to Knap et al., 2012) |
| Markovinovic2016 | nested RT-PCR (reference to Puchhammer-Stöckl et al., 1995) |
| Brockmann2018 | RT-qPCR (reference to Schwaiger et al., 2003) |
| Paulsen2019 | RT-PCR (reference to Andreassen et al., 2012) |
| Ilic2020 | RT-PCR (reference to Schwaiger and Cassinotti, 2003). |
| Wallenhammar2020 | Immunozym FSME IgG All Species (Progen Biotechnik GMBH, Heidelberg, Germany) |
| Blomqvist2021 | Immunozym FSME IgG All Species (Progen Biotechnik GMBH, Heidelberg, Germany), modified by the authors |
| Pautienius2021 | RT-PCR (reference to Schwaiger and Cassinotti, 2003). |
| Milonaki2022 | RT-qPCR (no details, no references to published protocols) |
| Gonzalez2022 | RT-qPCR (own developed protocol; reference to Gondard et al., 2018). |
| Paralikova2022 | Assay for the direct identification of the TBEv RNA but assay not specified (probably PCR; no details, no references to published protocols) |
| Malena2014 | Nested RT-PCR (no details, no references to published protocols) |

**Supplement S5.** Diagnostic tests used in the 16 included papers
