# Supplementary material for: A Systematic Review on the Prevalence of Tick‐Borne Encephalitis Virus in Milk and Milk Products in Europe
Source: Zoonoses Public Health. 2025 Feb 23;72(3):248–58. doi: 10.1111/zph.13216 (PMC11967290; doi:10.1111/zph.13216)
Supplement: Supplementary file 6 — Data S6. Critical appraisal of included studies. [file ZPH-72-248-s004.docx]

**Supplement S6. Critical appraisal of included studies.**

**Table 1 Critical appraisal of included studies – using the JBI case series tool**

| **Author, year** | **Question 1** | **Question 2** | **Question 3** | **Question 4** | **Question 5** | **Question 6** | **Question 7** | **Question 8** | **Question 9** | **Question 10** | **Total** |
| --- | --- | --- | --- | --- | --- | --- | --- | --- | --- | --- | --- |
| Khol1996 | Y | Y | Y | NA | Y | N | NA | NA | Y | Y | 6 |
| Holzmann2009 | Y | Y | Y | NA | Y | N | NA | NA | Y | Y | 6 |
| Caini2012 | Y | Y | U | NA | Y | N | NA | NA | Y | Y | 5.5 |
| Hudopisk2013 | N | Y | Y | NA | N | N | NA | NA | N | Y | 3 |
| Markovinovic2016 | N | Y | Y | NA | N | N | NA | NA | N | Y | 3 |
| Brockmann2018 | Y | Y | Y | NA | Y | Y | NA | NA | Y | Y | 7 |
| Ilic2020 | Y | Y | Y | NA | Y | Y | NA | NA | Y | Y | 7 |
| Mylonaki2022 | N | Y | Y | NA | N | N | NA | NA | N | Y | 3 |
| Gonzalez2022 | Y | Y | Y | NA | Y | Y | NA | NA | Y | Y | 7 |
| Paralikova2022 | N | Y | Y | NA | U | N | NA | NA | N | Y | 3.5 |

Question 1: Were there clear criteria for inclusion in the case series? Question 2: Was the condition measured in a standard, reliable way for all participants included in the case series? Question 3: Were valid methods used for identification of the condition for all participants included in the case series? Question 4: Did the case series have consecutive inclusion of participants? Question 5: Did the case series have complete inclusion of participants? Question 6: Were there clear reporting of the demographics of the participants in the study? Question 7: Were there clear reporting of the clinical information of the participants? Question 8: Were the outcomes or follow up results of cases clearly reported? Question 9: Was there clear reporting of the presenting site(s)/clinics(s) demographic information? Question 10: Was statistical analysis appropriate? Y=yes, N=no, U=unclear, NA= not applicable

**Table 2 Critical appraisal of included studies – using the JBI prevalence tool**

| **Author, year** | **Question 1** | **Question 2** | **Question 3** | **Question 4** | **Question 5** | **Question 6** | **Question 7** | **Question 8** | **Question 9** | **Total** |
| --- | --- | --- | --- | --- | --- | --- | --- | --- | --- | --- |
| Cisak2010 | U | Y | N | N | Y | Y | Y | Y | NA | 5.5 |
| Paulsen2019 | U | Y | N | Y | Y | Y | Y | Y | NA | 6.5 |
| Wallenhammar2020 | Y | Y | U | Y | Y | Y | Y | Y | NA | 7.5 |
| Blomqvist2021 | Y | Y | Y | N | Y | Y | Y | Y | NA | 7 |
| Pautienius2021 | Y | Y | U | Y | Y | Y | Y | Y | NA | 6.5 |
| Malena 2014 | U | Y | N | Y | Y | Y | Y | Y | NA | 6.5 |

Question 1: Was the sample frame appropriate to address the target population? Question 2: Were study participants sampled in an appropriate way? Question 3: Was the sample size adequate? Question 4: Were the study subjects and the setting described in detail? Question 5: Was the data analysis conducted with sufficient coverage of the identified sample? Question 6: Were valid methods used for the identification of the condition? Question 7: Was the condition measured in a standard, reliable way for all participants? Question 8: Was there appropriate statistical analysis? Question 9: Was the response rate adequate, and if not, was the low response rate managed appropriately? Y=yes, N=no, U=unclear, NA= not applicable
